# Nursing Care Related with Surgical Position*

**DOI:** 10.17533/udea.iee.v41n1e03

**Published:** 2023-03-14

**Authors:** Ángela María Salazar Maya, Sandra Patrícia Osorio Galeano

**Affiliations:** 1 Nurse. PhD in Nursing. Full Professor, Faculty of Nursing, Universidad de Antioquia and Universidad CES. Colombia E-mail: angela.salazar@udea.edu.co. Universidad de Antioquia Faculty of Nursing Universidad de Antioquia Colombia angela.salazar@udea.edu.co; 2 PhD. Associate Professor, Faculty of Nursing, Universidad de Antioquia. Colombia. E-mail: sandra.osorio@udea.edu.co Universidad de Antioquia Faculty of Nursing Universidad de Antioquia Colombia sandra.osorio@udea.edu.co

**Keywords:** surgical procedures, operative, patient positioning, nursing care, procedimientos quirúrgicos operativos, posicionamiento del paciente, atención de enfermería, procedimientos quirúrgicos operativos, posicionamiento del paciente, atención de enfermería

## Abstract

The patient’s correct position is necessary to conduct a safe and effective surgical procedure. This position depends on the access route, duration of the procedure, kind of anesthesia, devices to use, among other factors. This procedure requires planning and effort by the surgical team where they share responsibility to establish and maintain the correct positions for patients. Each surgical position fulfills an objective and implies risks to patients, which is why nursing professionals must be very attentive to provide the necessary care and ensure reliable practices in each position during the perioperative, the importance of the documentation, and the NANDA, NIC, and NOC taxonomy to consider.

## Resumen

La correcta posición del paciente es necesaria para el desarrollo de un procedimiento quirúrgico seguro y eficaz. Esta posición depende de la vía de acceso, la duración del procedimiento, el tipo de anestesia, los dispositivos a utilizar entre otros factores. Este procedimiento requiere planeación y esfuerzo del equipo quirúrgico donde se comparten responsabilidad para establecer y mantener las posiciones correctas para los pacientes. Cada posición quirúrgica cumple un objetivo e implica un riesgo para el paciente, por lo que los profesionales de enfermería deben estar muy atentos para brindar los cuidados necesarios y asegurar prácticas confiables en cada posición durante el perioperatorio, la importancia de la documentación y la taxonomía NANDA, NIC y NOC a tener en cuenta.

## Resumo

A posição correta do paciente é necessária para o desenvolvimento de um procedimento cirúrgico seguro e eficaz. Essa posição depende da via de acesso, da duração do procedimento, do tipo de anestesia, dos aparelhos a serem utilizados, entre outros fatores. Este procedimento requer planejamento e esforço da equipe cirúrgica onde a responsabilidade é compartilhada para estabelecer e manter as posições corretas dos pacientes. Cada posição cirúrgica cumpre um objetivo e implica um risco para o paciente, por isso os profissionais de enfermagem devem estar muito atentos para prestar os cuidados necessários e garantir práticas confiáveis em cada posição durante o período perioperatório, a importância da documentação e taxonomia NANDA, NIC e NOC para levar em conta.

## Introduction

The Royal Academy of the Spanish Language defines safety as "the quality of insurance that is free and exempt from all danger, harm or risk".^1^ From psychology, Abraham Maslow, American psychologist, developed theoretically the hierarchy of needs, which he ranks from physiological to self-realization; that in second place is physical safety, health, moral, family, employment, resources, private property.^2^ In nursing, its pioneer, Florence Nightingale, referred to safety as “all external conditions and influences that affect the life and development of an organism are able to prevent, suppress, or contribute to disease and death”.^3^ In turn, Virginia Henderson identified 14 basic human needs, and that in ninth place is defined as: “avoid dangers in the environment and avoid injuring others”^4^ on which she based nursing care. Faye Glenn Abdellah formulated 21 nursing problems, the third of these defined it as: “promote safety by preventing accidents, injuries, or other trauma and preventing the spread of infection”.^4^

Prevention of injuries through position is essential for the patient’s safety in the perioperative setting. It is important to know the risk factors and safety precautions that must be taken to avoid them.^5^ Likewise, identifying patients who are at risk, given that they can be subjected to intense pressure during the surgery, above all in the bony prominences because due to the position, they are exposed to friction or shearing during the transfer to the operating table and generally, suffer significant comorbidities.^6^ In addition to the aforementioned, they are under the effects of sedation or anesthesia, so they depend on the members of the surgical team who will be their advocates during the procedure. Sometimes, patients are not placed properly to distribute their body weight uniformly and this leads to greater risk of tissue damage. 

The objectives of positioning the patients include stabilizing them to keep them from moving;^7^ maintaining their comfort, privacy, and dignity, avoiding undue exposure;^8^ providing adequate exposure of the surgical site, ensuring ventilation and a patent airway; providing adequate access to intravenous lines, diuresis, blood loss, irrigation, drainage bags, visibility of measuring devices; watching for and protecting fingers, toes, muscles, nerves, and bony prominences from injury. Although the election of the position for a given procedure rests with the surgeon, the decision must be made among anesthesiology, nursing, and other staff of the surgical team. Nursing, as the patient’s advocate, must question any position chosen if it compromises the patient’s health and safety.^9^ Hence, this article addresses the risk factors that should arise during a surgical procedure for the nursing staff to keep them in mind during the preoperative, intraoperative, and postoperative, especially when placing the patient in the different surgical positions, like supine, prone and its variants, lateral, among others; as well as the importance of the documentation and interventions and goals to care for these patients. 

## Risk factors

Among the risk factors during the intraoperative, there are rubbing against surfaces, shearing, multiple surgeries, long times of surgical procedures, different supports and attachments for the position, vasoconstrictor medications and those used in anesthesia and sedation, instruments, and some surgical specialties, hypothermia and hypotension.^6^ The pad on the operating table may alter the risk of developing a pressure lesion, for this reason, the recommendations of the National Pressure Ulcer Council must be followed, which defines a support Surface as “a specialized device for the redistribution of pressure, designed for management of tissue loads, microclimate and/or other therapeutic functions”.^10^ The characteristic of a pad’s essential safety is to redistribute pressure, especially on the patient’s bony prominences.^6^ The postoperative period includes the use of vasopressors, mechanical ventilation, administration of sedative medications, postoperative use of corticosteroids, length of stay > 3 days, and extended hours in the intensive care unit.^6^

## Nursing care

### Preoperative nursing care

All perioperative patients have some risk of developing an injury, even during short procedures. Thereby, they must be evaluated carefully before surgery and identify risk factors to plan the implementation of their care.^6^ The evaluation of the skin includes temperature, edema, reddening, changes in skin consistency, and pain; it is necessary to document them in the clinical chart, as well as additional measures to take as the case may be. According to Spruce,^6^ the most common sites for pressure lesion are the sacrum (70%), heels (12%), and chin, sternum and trochanters (6%). Lesions on the sacrum and heels are associated with the supine position and those on the chin, sternum, and trochanters are associated with the prone position.^11^

In addition, it is important to assess the type and estimated duration of the procedure, the patient’s capacity to withstand the intended position, the surgical exposure required, the potential change of position, positioning devices required, comorbidities, the patient’s age, the body mass index, and use of surgical tourniquet.^8^^,^^12^ Another risk factor is the prone decubitus or Trendelenburg position due to postoperative loss of vision;^12^ in this case, it is important to inquire regarding preoperative anemia, vascular conditions, obesity, smoking, elderly adult, male sex, and diabetic retinopathy. Similarly, it is fundamental to keep in mind other risk factors, like spinal cord injuries, previous pressure injuries, skin problems (blistering, bruising, redness) in areas at risk of pressure lesions (sacrum, heels, occiput, bones, prominences), hemodialysis, creatinine level > 3 mg/dL, albumin level > 3 g/dL, limited mobility, fecal incontinence, anemia, malignancy, low weight, presence of pain or inhibited sensation of pain, low hemoglobin level, infections, poor nutritional status, and ASA of ≥ 3.^6^

Until recently, the only scale to evaluate the perioperative risk of pressure lesion was Braden’s Scale; currently, there are two scales to evaluate the risk of ulcer for perioperative patients: the Scott Triggers tool and the Munro Scale designed to identify risk in this population.^11^ The Scott Triggers tool evaluates age, albumin or body mass index, ASA score, and estimated duration of the surgery to determine if the patient has a high risk of pressure lesion. Use of this scale requires authorization.^13^ In turn, the Munro Pressure Ulcer Risk Assessment Scale^14^ uses a cumulative score to evaluate factors during the preoperative, including “classification of anesthesia, method of anesthesia, intraoperative body temperature, hypotension, skin moisture, posture change during surgery, and surgical posture. A total score of ≤13 indicates low risk of pressure ulcers”.^15^ It also evaluates, principally, operation time and blood loss; a total score of ≤15 indicates low risk of pressure ulcers.^15^

Patients going into surgery must remove jewelry, piercings, false hair, accessories or other items that may pose a risk for positioning injury. All equipment should be checked prior to inducing the anesthetic, ensuring that the frame or rollers are appropriate for the patient’s size. Armrests, head support, and all extra padding should be near.^16^ Patients for orthopedic procedures tend to require specialized tables and special equipment; in such case, it must be ensured that the equipment or tables necessary for the procedure are available, correctly configured and verified with the surgeon before the patient enters the operating room. Also, It must be inspected for its proper functioning, its cleanliness and that it is ready to be used with the patient. 

### Nursing care during the intraoperative

Practices when positioning the patient include protecting the patient's eyes and it is important with all general anesthetics; it is usually accomplished by using of adhesive tape or a transparent dressing.^16^ The perioperative staff must consider using supports for high-risk patients. Sheets and blankets should not be used because they diminish the effectiveness of the support surfaces and can cause pressure; In addition, they must be free of wrinkles and the patient must be free of moisture caused by skin preparation or other sources.^6^ Regarding supports, it is important to determine the equipment and devices that will be used for the position, according with the surgeon’s indications and the risk factors that may arise; confirm the availability of equipment required for the position when the procedure is scheduled; use equipment and devices that support the weight and size of the patient, as well as the different articulations necessary to safely move the patient; verify the cleanliness, surface integrity, and correct operation of equipment, devices, and support surfaces. Use equipment and devices according with the manufacturer’s instructions and verify compatibility between positioning devices and support surfaces prior to using them.^12^

Independent of the position, bony prominences and high-pressure areas must be protected. Additional protection measures depend on the patient’s own risks, type of surgery and duration, position, and policies of the surgical center.^6^ When moving patients to and from the operating table or when the position is being accommodated, it is suggested not to slide or pull as it may result in shear forces or friction on the patient's skin.^6^

Damage to the skin can occur if patients are pulled without support or when sheets are used to move them and friction is produced when the skin rubs between surfaces. To avoid these injuries, a lateral transfer device should be used, which is a sliding table that adequately supports the patient during transfer to or from the operating table.^8^

The Association of periOperative Registered Nurses (AORN)^12^ recommends the following aspects to provide a proper position:

- Plan the position and evaluate risks, needs, and requirements.
- Determine proactively the person responsible for assisting the patient while on the operating table to prevent falls.
- Determine the regular intervals in which the patient’s position must be revised, along with safety straps, devices and equipment during the surgery to check that the patient is placed correctly and has not had any movement.
- Communicate presence of critical devices (urinary catheters, drainage tubes), secure them during the positioning and confirm their patency.
- Keep the head and neck in neutral position without extreme lateral rotation or hyperextended for long periods.
- Protect the patient’s eyes.
- Maintain the patient’s body aligned physiologically.
- Avoid the body’s contact with the operating table’s metal surfaces and make sure the patient’s limbs do not fall or hang below the table’s level.
- Place patient’s arms adequately.
- Watch the position of the hands, feet, and toes during positioning, even when conducting changes in the operating table.
- Evaluate the patient’s pulse after fastening the safety straps to verify adequate perfusion.
- Supervise the patient’s position during the procedure and take indicated corrective measures.
- Implement repositioning interventions to redistribute pressure, if possible.
- Verify that no equipment or devices are on the patient.
- Revise the effectiveness of the positioning interventions during the postoperative.

The AORN also establishes that special considerations must be had with the following groups of patients:

- *Pregnant women:* when subjected to obstetric surgery, their position is with a left lateral inclination, placing a device under the right lumbar region above the iliac crest and below the lower costal region in a 12-cm wedge shape, until reaching a lateral inclination from 12 to 15 degrees. Also, a pillow can be placed under the right pelvis to achieve a 12- to 15-degree lateral inclination, or tilt the surgical bed 15 to 45 degrees to the left. Likewise, if the woman will undergo non-obstetric surgery and is > 16 weeks pregnant, she must have left lateral inclination during the operative procedure.^12^
- *People with obesity*: select the operating table indicated for patients with morbid obesity; an obese patient with a body mass index < 40 kg/m^2^, must be placed in Trendelenburg position, raising the patient’s head from 25 to 30 degrees, elevating the operating table or using a device that holds the patient’s head and shoulders. Estimate using a device under the patient’s right lumbar region or a 15-degree left inclination of the operating table; use padded arm protectors to contain the patient's arms at the sides of the body and in Fowler or semi-Fowler position; keep the abdominal pannus from resting on the thighs, and if possible, place foam padding between them.
- *Pediatric patient:* in this population, it is important to consider that the younger the age, the greater the risk of skin lesions derived from inadequate positioning during the surgery. Newborns prior to the 37 weeks of gestation have an immature stratum corneum, which implies structural differences on the skin compared with the pediatric and adult population.^17^ For newborns, it is recommended to place cotton or gel pads on the pressure points, avoid compression and inadvertent elongation of the vascular-nervous bundles, protect the eyes, fix the orotracheal tube and the respiratory circuit. The prone position is common for corrections of neurological congenital malformations and if there is pressure on the abdomen due to an inadequate position, ventilation is altered, compressed vena cava and increased epidural venous pressure, bleeding, and edema on the tongue and face. The Fowler or semi-Fowler position is used for posterior fossa access in children older than four years, although it reduces intraoperative bleeding and facilitates surgical exposure, hemodynamic instability, venous air embolism, and postoperative pneumocephalus may occur.^18^^,^^19^

Multiple strategies exist to maintain a proper posture and, thus, avoid injury, such as the use of rolls, containment nests, maintain a physiological posture, and individualized care according to the newborn’s characteristics. The newborn’s position for surgery depends on the kind of surgical approach; however, before covering the child with the surgical fields, it is important to evaluate limb perfusion, through the control of capillary refill and extremity heat.^20^

### Nursing care during the postoperative

It is fundamental for nurses to evaluate patients in search of skin and musculoskeletal injury. They must evaluate patients in search of signs of intraoperative injury and must inspect any area identified as high risk of injury during the preoperative evaluation. The evaluation must be included in the report of transition of care to the post-anesthetic unit.^9^ Unfortunately, pressure lesions, often, are not immediately identified, but up to 72 h after when signs of injury appear.^21^ For this reason, during the postoperative an immediate evaluation must be conducted to identify any change in the skin in comparison with the preoperative evaluation of the skin; besides defining criteria to identify pressure lesions.

If there are no visible signs of injury, it is still important to report the evaluation of the skin to the staff assuming the postoperative care so they can be diligent in identifying injuries during the postoperative.^22^ The staff in the unit attending the patient during the postoperative must notify the perioperative department if a pressure lesion appears within 72 h after surgery because it may be attributed to the perioperative period. In this regard, high-risk patients who are outpatients and are discharged to their homes must receive instructions on what to look for and what to report to the health staff if a pressure lesion appears.^22^

The perioperative staff should ask patients about their skin during the postoperative follow up, conducted through phone calls to identify if an additional evaluation is needed and organize follow-up care if necessary.^22^ Lesions discovered in the post-anesthesia care unit are more likely to occur during the intraoperative, but those with a late onset (up to 7 days) are likely to have occurred during the late postoperative period.^16^

## Surgical positions

### Supine position

**Figure 1:**
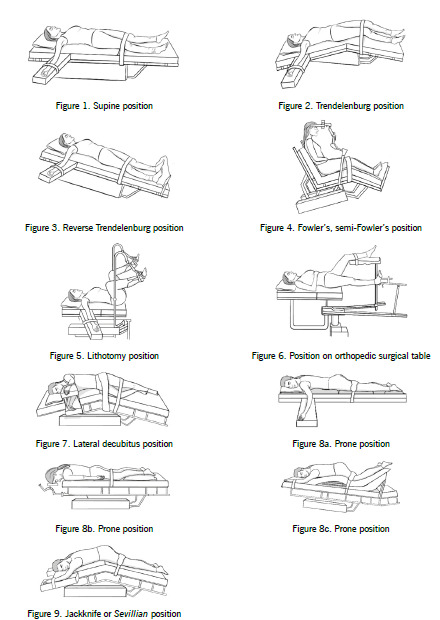
Credits: all the figures were drawn by Carolina Mazo Ávila

It is also known as decubitus dorsal. In this position, the body rests on the back, the head is placed on a small pillow, the upper limbs may be on the sides or parallel to the body and the lower limbs are extended, depending on the surgical procedure. To maintain the position, the arms are held, keeping them from slipping and preventing compression of the neurovascular bundles (Figure 1). The back is protected with a mattress to avoid injury to the tissues that cover the heels, the sacrum, the scapulae, and the occipital region. Patients are anesthetized in this position and then are repositioned, if necessary. It is most-often used for procedures requiring access to the front of the body: general surgery, reconstructive and plastic procedures, which involve the anterior part of the thorax, the epigastrium, and the pelvis. This position is also used for hand, forearm, knee, foot, anterior cranial and cervical structure interventions and patients with multiple injuries who require simultaneous procedures or those requiring intramedullary nailing of tibial fractures.^16^

When patients assume this position, they experience a decrease in heart rate, in vascular resistance, in functional residual capacity and total lung capacity. Further, it provokes an increase of pressure on dependent skin over the sacrum, elbows, and heels. Ligaments in the spine are relaxed by anesthetic agents and can lead to back pain.^16^ Alopecia results from pressure on the occiput, especially in the presence of hypothermia and prolonged surgeries.^8^^,^^16^ When placing patients in the position, it is important to place a pillow under the knees to reduce back pain. Using padded cushions or foam pads under the patient's sacrum, elbows, heels, and occiput will increase the patient's overall comfort.^16^ The arms must be placed on the sides with a sheet and be secured with the protectors or extended on the boards for this purpose, in abduction < 90°,^8^ with palms up, and gently secured to the arm board. If the arms are parallel to the body, the sheet should extend from the upper half of the arm to the tips of the fingers.^8^

Nerve damage is the second most-common type of anesthetic complication according to the American Society of Anesthesiology.^16^ Ulnar neuropathies are the most frequent, followed by those of the brachial plexus. Mechanisms for nerve injury include stretching, compression, ischemia, metabolic disturbance, and surgical injury, a given threshold of pressure or duration of the compression.^16^ Keep the patient’s arms from falling off the table or the armrest and coming into contact with the metallic part of the operating table; the arms must be parallel, the patient's ankles without crossing and elevating the patient's heels from the underlying surface with a device designed for this purpose and to distribute the weight of the patient's leg along the calf,^8^ place the safety strap, approximately 5 cm above the patient’s knees and protect the patient's feet from hyperflexion or extension.

Sometimes, lateral head rotation is required, leading to injury to the nerve bundles, caused by increased pressure and stretching of the brachial plexus.^23^ This risk also increases when the patient’s arm is abducted > 90°, hence, it is important to maintain neutral alignment of the head and arms with respect to the patient’s body.^8^ The possibility exists of injury to the radial and ulnar nerves due to compression against the edge of the armrest.^16^ For procedures on the head, neck, and thorax, the arms are placed at the side of the patient; elbows, ulnar nerve areas and hands are protected by foam padding and palms face in. A sheet is used over the arms and gently passed under the patient's body, keeping the arms from falling off the operating table with the risk of injuring nerves and tissues.^12^

Variations to the supine position will be discussed ahead:

1. Trendelenburg. This position is characterized by the head being below the horizontal line.^24^ The table is tilted at an angle between 10 and 30° so that the head is lower than the body. Patients are placed in this position when seeking to reject the abdominal contents cephalad. (Figure 2). It is useful for many surgical procedures, especially, surgeries of the lower abdomen and pelvic cavity, in maneuvers for the prevention and management of air embolisms to the brain during open-heart surgery, and when engorgement of the jugular vessels is desired to puncture and insert catheters in the subclavian and jugular vessels. Among the changes that occur, the elevation of arterial pressure stands out because venous return from the lower extremities to the right atrium is favored and, therefore, preload and cardiac output increase, and peripheral vascular resistance decreases. The foregoing can lead to cerebral venous congestion and conjunctival edema, especially when the head is lower than the rest of the body; respiratory function is moderately affected by decreased vital capacity in procedures lasting more than 90 min, although this is compensated with positive pressure ventilation in intubated patients. Acute glaucoma may occur due to increased intraocular pressure and elevated cerebral venous pressure is a risk for patients with brain metastases or predisposing factors. 

When placing patients in this position, measures should be implemented to mitigate the risk of increased intraocular pressure, like minimizing the degree of Trendelenburg as much as possible, implementing measures to keep the patient from slipping on the operating table, securing the arms without using braces or circumferential wristbands, and keeping the patient in this position for as short a time as possible. 

Use a padded platform to keep the patient from slipping and reduce the possibility of injuries to the peroneal and tibial nerves from flexion of the foot or ankle; monitor the patient’s feet and implement corrective measures.^12^

2. Reverse Trendelenburg position. The table is tilted in the opposite direction of Trendelenburg, leaving the head higher than the horizontal line. It is useful for surgeries of upper abdomen in gallbladder and stomach. It can be used for anterior approach to the cervical spine, radical neck dissections, carotid artery interventions and tracheostomy^25^ (Figure 3).

3. Fowler's or semi-Fowler's position. A variation of the supine position is the Fowler position (that is, seated position) or the semi-Fowler position (that, semi-seated or beach chair position).^16^ In the bent-knee section, the trunk is raised 40 degrees to bring the patient into a semi-sitting position. It is useful in patients suffering from heart or respiratory failure (Figure 4). After induction and intubation, the patient is positioned seated, with the head at the necessary height and with the body at variable degrees of lateralization; in all cases, place the head in the prone position and use metal frames with pegs to support the skull.^26^

In neurosurgery, an excellent exposure of the operating field is mandatory and it is necessary to keep the position of the skull fixed for the time required. For this, it is necessary to have the aid of gravity to optimize hemostasis and reduce intracranial pressure without the position having an impact on respiratory and cardiovascular dynamics. For this purpose, numerous positions have been devised to keep the head above the horizontal plane, and technological refinements to fix the bone plane and maintain the position during the execution of microscopic maneuvers.^27^

It is also used for orthopedic procedures requiring access to the shoulder. An advantage of this position for shoulder surgery is that, when performed laparoscopically, it allows the procedure to be in an open approach and is easier than if the patient were in the lateral decubitus position. To avoid cerebral hypoperfusion, the degree of elevation of the patient's head should be minimized as much as possible.^8^^,^^28^ When patients experience cerebral hypoperfusion, the brain is at risk of ischemic injury, which can cause cognitive impairment, organ damage, stroke, and death;^29^ it is also important to keep the patient’s head in neutral position without extension, flexion, or rotation.^8^ Hyperextension of the patient's neck could result in spinal cord injury,^30^ whereas rotation of the patient's neck could cause compression neuropathy or stretching of the glossopharyngeal, vagus, and hypoglossal nerves.^8^^,^^31^

4. Lithotomy position. Physiological changes vary according to the leg position and are mainly cardiac and respiratory. Elevating the legs results in increased cardiac output and venous return. Respiratory changes are caused by increased intra-abdominal pressure that limits the diaphragm’s movement, resulting in decreased lung volumes. These changes are more marked in exaggerated lithotomy and require ventilation to be controlled.^16^ The most vulnerable nerves in this position are common peroneal, obturator, saphenous, and femoral. The legs must be placed so that no part of the leg rests on the supports^16^ (Figure 5). 

Standard and low lithotomy positions are used mainly when the surgeon requires access to the perineum and abdomen and for gynecological procedures, sigmoid colectomy and genitourinary procedures, such as cystoscopy; the pelvis is raised and the legs are flexed higher on the trunk. This elevation stresses the lumbar spine and stretches the lumbosacral muscles and ligaments. Perfusion of legs and feet is reduced and the pressure of abdominal contents against the diaphragm is increased, requiring intubation and controlled ventilation.^16^

The exaggerated lithotomy position is used for procedures requiring trans-perineal access to the retropubic area, such as a perineal prostatectomy.^16^ If the procedure is prolonged there is a high incidence of compartment syndrome of the lower extremities. With legs flexed over the trunk, compression of the thigh muscles can cause edema and, because the fasciae of the muscles lack elasticity, any edema puts more pressure on the muscle tissue, reducing perfusion and increasing ischemia that potentially lead to necrosis. These ischemic muscles release large amounts of myoglobin into the blood from the injured cells, causing kidney damage and possible kidney failure.^16^^,^^32^

When the patient is in this position, it is essential to take into account that the patient's buttocks are at the lower level of the operating table so as to firmly support the sacrum; for this, it is necessary to pad the patient’s buttocks; ensure that the supports or stirrups are at the same height; avoid excessive flexion, rotation or abduction of the patient's hips; slowly and simultaneously place the legs on the stirrups (minimum one person per leg); placing additional padding around the patient's foot and ankle when using cane-shaped leg supports; keep the patient in lithotomy position for as short a time as possible; ensure that members of the surgical team do not lean on the patient's legs and do not place the safety restraint system on the patient's chest or abdomen. 

Arms can be placed on the armrests, with the palms of the hands facing up and softly secured, or parallel to the body and it is important to keep in mind that they can migrate over the edge of the operating table, so the risk of trauma to the toes is significant when the bottom of the bed is lifted. Arms should have elbows protected by a foam pad and positioned with palms facing the body, but hands should be secured within a foam pad to prevent fingers from slipping out.^16^ During the procedure, it is necessary to change the patient’s position at established intervals. It is recommended to keep the patient's head in a neutral position without excessive flexion, extension or rotation; at this point, a horseshoe shaped head positioner should not be used; flex and secure the patient's arms; flex the patient's knees 30°; verify the placement and security of the restraint system through the patient's thighs; protect hands and fingers from injury when the bed bottom is raised, lowered or repositioned. Before lowering the patient’s legs to the bed surface, remove supports from legs slowly and move them simultaneously (at least one person per leg). Various supports are available and selection is based on the patient's anatomy and range of motion.^16^

5. Hemi-lithotomy position. A common position used for patients undergoing fracture fixation, arthroplasty, or hip arthroscopy. This is a variation of the lithotomy position in which the patient is positioned on a fracture table with the leg in standard lithotomy and straightened in traction. The danger of placing a patient's non-operated leg in this position is that they develop compartment syndrome,^8^ which occurs when pressure is exerted on a muscle because the blood flow to the muscle diminishes, preventing oxygen and muscle nutrition; by relieving pressure, damage can be permanent with tissue necrosis. Patients are anesthetized on a stretcher, then are transferred to the fracture table, where the perineal post should be well padded and the patient's feet should also be padded, traction is released as soon as it is not needed and periodically release traction during surgery.^7^

6. Position on the orthopedic surgical table: it is a specialized positioning device used to position femoral fracture and some types of hip fractures. This operating table permits placing the fractured leg in traction so that bone fragments can be manipulated, realigned and fixed. The patient is anesthetized prior to being transferred to this place. The arm on the side of the fracture should be positioned securely across the patient's chest to allow the surgeon full access to the fracture. A vertical post is placed in the perineum. This cane should be well padded and is placed against the pelvis between the genitalia and the unaffected leg. Positioning it incorrectly can damage the genitals and pudendal nerves. Other complications include brachial plexus injury and compartment syndrome of the lower limbs^33^ (Figure 6). 

### Lateral decubitus

It is used for orthopedic procedures involving the hips or shoulders and, with some modifications, for renal and thoracic procedures. In this position, the patient is placed on the non-operated side. Patients run the risk of injury due to the pressure exerted on the dependent side in the ear, elbow, shoulder, iliac crest, hip, knee and ankle areas. It also places patients at risk of compartment syndrome and rhabdomyolysis.^7^ Prolonged surgery in this position can lead to vascular congestion and hypoventilation in the dependent lung. Hence, patients with pre-existing lung problems or with heart disease cannot tolerate this position (Figure 7).

To secure the position, it is necessary to have a sufficient number of people to turn the patient and prevent injuries, it is recommended to use this position for the shortest time possible; it is important to reposition the patient at established intervals to diminish the risk of compartment syndrome; place the head on a pillow or positioner; verify that the dependent ear is not bent; position the patient's arms on two levels, each arm on a board and abducted < 90°; place a roll under the thorax dependent on the patient, distal to the axillary fold at the level of the seventh to ninth rib, using a device designed for this; in this case, do not use rolled up sheets or towels; verify bilateral radial pulses after placing the roll; maintain the physiological alignment of the patient's spine. Likewise, a safety restraint system is necessary at the patient's hips, flexion of the patient's dependent leg at the hip and knee while keeping the upper part of the leg straight with a pillow placed between the legs to achieve stability in the position, place pads on the dependent knee, foot and ankle. ^7^^,^^16^

In renal or thoracic procedures, the lateral position is modified by flexing the bed to widen the intercostal spaces and improve thoracic exposure, the bottom of the bed is flexed with the chest remaining level. When access to the retroperitoneal area is required, the upper part of the bed is also flexed and the "kidney rest" is raised^16^ to increase flexion and improve exposure. The patient should be positioned so that the device is below the dependent iliac crest. If the renal support is elevated and aligned below the patient's flank, ventilation of the dependent lung is severely restricted.^16^

Blood pooling in the lower extremities occurs to varying degrees in all lateral positions and is greatest when patients are flexed. The use of compression stockings will help to minimize the systemic effect. In all lateral positions, the dependent lung receives more blood flow and it is easier to ventilate the upper lung. This effect is known as ventilation-perfusion mismatch. The presence of pre-existing heart or lung disease decreases the patient's ability to tolerate all these physiological changes.^16^ Although the surgical procedure defines the patient’s position, the surgeon’s preference also influences, for example, hip procedures can be conducted in lateral or supine position, but the surgeon’s skill and comfort determine the most suitable position for the procedure. Similarly, the position is usually a balance between what patients can physically handle and what they tolerate physiologically.^16^

### Prone position

The prone position is one of the most challenging positions for the perioperative team because placing the patient requires coordination among the team members, and particularly, special attention should be paid to safety. The additional challenge lies in that most patients are intubated and under general anesthesia when placed in this position.^9^ It is used to provide access to the back of the head in spinal procedures (including the cervical spine), to access posterior cranial structures, certain orthopedic procedures (Achilles tendon repair), and some rectal procedures. It is also used to replace the full sitting position.^7^^,^^16^ It is important to consider that this position exerts pressure on the abdomen, reducing blood flow through the inferior vena cava, causing congestion of the paravertebral and epidural veins, increasing bleeding in the surgical area along with a decrease in arterial pressure and hypovolemia. The foregoing may cause decreased perfusion to major organs and increase risk of acute kidney injury.^34^ For this reason, the patient's abdomen must be free of pressure because the abdominal content restricts the movement of the diaphragm^16^ and causes distension of the epidural veins, causing intra-abdominal pressure to increase venous congestion; to minimize this, frames and various methods have been developed to support the patient's abdomen, each with its own merits.^16^

Before placing a patient in the prone position, anesthesia is induced and the patient is intubated, after securing the endotracheal tube, and shielding the patient's eyes. Additional monitoring is performed of intravenous and arterial lines; an esophageal stethoscope and a temperature monitor and urinary catheterization. The turning and positioning sequence must be known by all participants in this process.^16^ Many devices are available to place a patient in prone position from different attachments to support the patient's shoulder and below the iliac crest to adjustable frames, such as Wilson’s, which is used for patients in a traditional prone position and Andrews’ for patients in kneeling position. These have to be well padded and adapted to the patient's body and it is the surgeon who chooses the kind of frame to use^16^ (Figures 8a, 8b, and 8c). 

Although the patient's head can be rotated to the right or left side, it is better if it is aligned to the midline. Many devices on the market avoid external pressure on the eyes and make it easier to keep the endotracheal tube clear, allowing a view of the patient's face and eyes. If the patient is to be placed with the head turned to one side, a soft gel or sponge support should be used, ensuring that the dependent eye is free of pressure and that the ear is well positioned and cushioned.^16^

The position of the arm in prone decubitus is chosen based on several factors. For procedures requiring a fluoroscope, the patient's arms should be secured at the sides, in line with the body, elbows and hands protected and secured with foam pads and held in functional position. If the arms are placed on boards, several options are available, such as the armrest parallel to the operating table. The arms should be placed on the padded boards, ensuring that no muscles are under tension, the elbow is free of any source of pressure, and the forearm, wrist, and hand are neutral and in line. To avoid pressure from the humeral head on the axillary neurovascular complex, the arms should not go above the head. After positioning the arms, the pulse should be checked at both wrists.^16^

If patients lie directly on their abdomen, lung volumes decrease due to impeded movements of the diaphragm. When the patient is in the prone position with support, the abdomen is free of pressure, lung compliance is near normal, and loss of functional residual capacity is less than in the supine or lateral positions. As stated by O’Connell,^16^ since 2006, “the prone position has been studied as a method to "recruit" collapsed alveoli and improve gas exchange in acute respiratory distress syndrome (ARDS)”, a position widely used in these times of COVID pandemic.

In prone decubitus, there is extravascular fluid accumulation in any dependent extremity, including the hands, feet, face, and conjunctiva. The longer the procedure, the more dramatic the edema. This can exacerbate certain pre-existing conditions, like intermittent numbness in the hands and will also affect the nose and pharynx. Therefore, patients who have had prolonged procedures or those who have questionable airways can remain intubated during the immediate postoperative period.^16^

One of the most-common physiological changes accompanying the transfer from the supine to the prone position is hypotension, which frequently occurs with induction of anesthesia caused by loss of autonomic tone and can be exacerbated by switching to the prone position. This effect can be more profound when using the Andrews frame because the legs are in a dependent position.^16^ Patients undergoing prolonged procedures in prone decubitus are vulnerable to pressure injuries to the skin, forehead, and chin that must be protected from excessive pressure. The knees, lower costal margins, and iliac crests are the most susceptible areas to pressure effects in this position. In addition to the pressure in these areas, the small movements that occur with the surgical devices can cause friction on the patient's skin.^16^

Ophthalmological problems after surgery in prone position^16^ range from loss of visual acuity to complete blindness; this is due to occlusion of the central retinal artery,^34^ anterior ischemic optic neuropathy, posterior ischemic optic neuropathy, and cortical blindness.^35^ Risk factors include: ^(^^16^ hypotension, anemia, blood loss, fluid management, diabetes, prolonged time in this position > 6.5 h, adverse drug effects, and anatomical variations in optic nerve blood supply. Most cases are associated with prolonged spine procedures under general anesthesia, posterior lumbar fusions, and scoliosis correction. This is related to a combination of blood loss, hypotension, and increased orbital venous pressure.^34^ The prone and Trendelenburg positions can elevate orbital pressure, causing decreased tissue perfusion to the eye.^34^^,^^35^ To diminish risks in this position, it is recommended to place the patient in some degree of inverted Trendelenburg to prevent pressure on the patient's face, maintain blood pressure, perform invasive hemodynamic monitoring, limiting blood loss and decreasing the duration of the anesthetic and surgical procedure.

There is a variation of the decubitus position, it is the Jackknife or *Sevillian*. It is used to provide exposure to the sacral, rectal and perineal areas. When operating under general anesthetics, the patient's chest is elevated using rolls or pillows when the patient receives regional anesthesia. Place a pillow under the patient’s hips, then the bed is flexed with the feet placed on a pillow to protect the toes. This position provokes the following physiological changes: cardiac, respiratory, and circulatory related with head and leg dependence. When the abdomen and thorax are raised from the surface of the bed, the head-down position pushes the abdominal viscera against the diaphragm, compromising breathing (Figure 9).^16^

It is essential to keep in mind not using positioning devices that place the head below the heart; avoid direct pressure on the patient’s eyes; place the head in neutral position without excessive flexion, extension, or rotation; do not use a horseshoe-shaped headrest and, if the head is in the midline, use a facial positioner and inspect the position of the face.^12^ Place the patient’s arms according to the needs of the surgical team and the patient’s physical limitations; make sure chest supports extend from the clavicle to the iliac crest to allow for full lung and abdominal expansion; free breasts, abdomen, and genitals from torsion or pressure; protect knees; check that toes are not in contact with the stretcher; place pads under the patient's shins; evaluate the pedal pulses; keep the patient in the prone position for the shortest time possible; and have the stretcher easily accessible in case of emergency. 

Perioperative nurses are the voice of patients and advocate for best practices for positioning, they are in charge of providing confidence and ensuring the best care possible, protecting patients from injury, and of possible litigation if a postoperative injury occurs.^9^ A strategy to ensure successful positioning is to work in collaboration with the surgeon and anesthesiologist. Training and verification of competencies ensure that perioperative staff understand how to correctly position patients when receiving positioning and procedures information. When placing the patient in prone position, the nurse must take measures to prevent lesions, document all actions carried out during the positioning, and evaluate the patient in search of any sign of injury after the operation.^9^

## Documentation

Interventions related with positioning must be documented, providing a description of the perioperative nursing care administered, state of the patient’s results in the transfer and information for the continuity of patient care. It is important to record in the clinical chart the preoperative evaluation, identity and titles of the people participating in positioning the patient, the patient’s position, including the location of arms and legs and any repositioning activity, type and location of the equipment or devices, of safety restrictions and any additional padding, specific actions taken to prevent injury to the patient, and the postoperative evaluation.^9^^,^^12^

## NANDA, NIC, NOC taxonomy

The Nursing Outcomes Classification (NOC), Nursing Interventions Classification (NIC) (independent and interdependent) and the standardized language of nursing, allow care during the perioperative. To summarize, the NANDA taxonomy proposes the nursing diagnosis: “[00087] Risk of perioperative postural injury, approved in 1994 and revised in 2006, 2013, 2017, 2020”. Defined as: “Susceptible to inadvertent anatomical and physical changes as a consequence of the posture or equipment used during an invasive surgical procedure that may compromise health”.^36^

*The characteristics and related factors are in:* dehydration, inadequate access to appropriate equipment, inadequate access to adequate support surfaces, inadequate availability of equipment for people with obesity, malnutrition, prolonged non-anatomical posture of the extremities, rigid support surface, people at age extremes; people in lateral, lithotomy, prone, Trendelenburg positions; people undergoing a surgical procedure > 1 h, diabetes mellitus, general anesthesia, immobilization, neuropathy, sensory-perceptual alterations due to anesthesia, vascular disease.^36^Table 1 presents NANDA 00087 Diagnosis with its respective goals and related interventions. 

**Table 1:** Risk of perioperative postural injury - NANDA 00087 diagnosis

NOC Related	NIC Related
[1101] Tissue Integrity: Skin and Mucous Membranes: Structural integrity and normal physiological function of the skin and mucous membranes	[842] Change of position: intraoperative
[1913] Severity of physical injury: Severity of signs and symptoms of bodily injury.	[3590] skin surveillance
	[4062] Circulatory care: arterial insufficiency
	[4066] Circulatory care: venous insufficiency
	[4028] Decreased bleeding: wounds
	[6610] Risk identification
	[2660] Management of altered peripheral sensation
	[4120] Liquid management
	[2550] Improved brain perfusion
	[4070] Circulatory precautions
	[4110] Embolism precautions
	[2920] Surgical precautions
	[3902] Temperature regulation: perioperative
	[6650] Surveillance
	[764] Care of the cast patient: wet cast
	[6486] Environmental management: safety
	[7980] Incident report
	[6680] Monitoring of vital signs
	[6490] Fall prevention

## Final considerations

Although many aspects of the patient’s positioning are fundamental, it is recommendable to establish guidelines and procedures in the surgical center and contribute for the staff to develop skills to ensure proper placement. 

Safe and comfortable positioning is a priority in surgery; due to this, ensuring that each member of the operating room team is aware of the scope of the possible problems of positioning will avoid adverse events. The practices recommended in this article are achievable in nursing praxis. Preventing damage from incorrect position requires anticipation of the entire equipment necessary to provide the position and plan the procedure that will be performed, application of ergonomic principles and of body mechanics. Special attention must be paid to the patient’s comfort and safety, as well as to the evaluation of the circulatory, respiratory, integumentary, and musculoskeletal systems and neurological structures. Working as part of a team can minimize the risk of perioperative complications related with the position.
